# Is olive crop modelling ready to assess the impacts of global change?

**DOI:** 10.3389/fpls.2023.1249793

**Published:** 2023-11-27

**Authors:** Francisco J. Villalobos, Álvaro López-Bernal, Omar García-Tejera, Luca Testi

**Affiliations:** ^1^ Instituto de Agricultura Sostenible, Consejo Superior de Investigaciones Científicas (IAS-CSIC), Córdoba, Spain; ^2^ Departamento de Agronomia, ETSIAM, Universidad de Córdoba, Córdoba, Spain; ^3^ Departamento de Ingenieria Agraria y del Medio Natural, Universidad de La Laguna, San Cristobal de La Laguna, Spain

**Keywords:** climate change, crop simulation model, phenological development, photosynthesis, *Olea europaea* L., water use

## Abstract

Olive trees, alongside grapevines, dominate the Mediterranean tree crop landscape. However, as climate change intensifies, the Mediterranean region, which encompasses 95% of the global olive cultivation area, faces significant challenges. Rising carbon dioxide (CO_2_) levels, increasing temperatures, and declining precipitation pose substantial threats to olive tree performance. Photosynthesis, respiration, phenology, water use and ultimately yield are possibly the main factors affected. To address this future scenario, it is crucial to develop adaptation and mitigation strategies. Nevertheless, breeding programs and field management practice testing for tree crops are time-consuming endeavors. Fortunately, models can accelerate the evaluation of tailored solutions. In this review, we critically examine the current state of olive tree modeling and highlight key areas requiring improvement. Given the expected impact of climate change, prioritizing research on phenology, particularly regarding bloom and pollination, is essential. Simulations of biomass should incorporate approaches that account for the interactive effects of CO_2_ and temperature on photosynthesis and respiration. Furthermore, accurately simulating the influence of water stress on yield necessitates the development of models that integrate canopy behavior with root performance under conditions of water scarcity. By addressing these critical aspects, olive tree models can enhance our understanding of climate change impacts and inform sustainable agricultural practices.

## 1 Introduction

The cultivation of olive (*Olea europaea* L.) trees started somewhere in the eastern Mediterranean area long before the Greek and Roman civilizations, at around 4000 BC (Kostelenos and Kiritsakis, 2017). As an appreciated source of food and oil, olive trees then spread over centuries around southern Europe and northern Africa. Only recently, olive growing has been introduced to other areas of the world, but more than 95% of the 10 Mha covered by olive orchards is still concentrated in Mediterranean basin countries (FAOSTAT, 2023). Historically a low input crop grown on rolling landscapes with shallow soils, olive trees withstand the harsh drought-prone conditions prevailing in most olive growing regions. The capacity of olive cropping systems to grow and yield even under these unfavorable conditions is related both to several physiological traits of this species and to appropriate management schemes (Connor, 2005), and it has been key for the wide expansion of the crop in regions with a Mediterranean climate.

Traditional techniques of olive production, established by trial and error in the absence of thorough scientific understanding, have persisted for centuries. Since water deficit is the main factor limiting yield expression, critical aspects of management target the control of tree transpiration and non-consumptive water losses. As a result, traditional rainfed olive orchards exhibit low planting densities (usually below 160 trees ha^-1^) and small canopy size, with soil management oriented to minimizing water use by the understory through tillage, mowing, grazing or herbicides. New, more productive, olive cropping systems have appeared in the last decades due to the introduction of irrigation and technological innovations. They are characterized by narrower tree spacings (even >1500 trees ha^-1^ for hedgerow orchards) and higher use of inputs (water, fertilizers, pesticides), being suitable for mechanical pruning and harvesting in many cases.

Olive has special features that make it especially hard to analyze in terms of response to climate change and adaptation. First, most olive orchards concentrate around the Mediterranean basin, where changes in rainfall and temperature are expected to be more dramatic than in other areas (Simolo et al., 2014; IPCC, 2021). Forecasts for the worst scenarios show an increase in average temperatures from 2 to 5 °C and precipitations reduced by 10-30% by the end of the century in many Mediterranean olive growing areas. Second, being a perennial tree there are aspects of its physiology that may require new models (e.g. use of reserves, mortality). Gaps in knowledge are wider in trees in general and in olives in particular due to limited research funding. In addition to that, the time scale is much longer than in annual crops, breeding programs are slower, and management decisions take years to have an effect and have to consider the expected conditions of the next 10 to 20 years.

In this review, we want to analyze if available modelling tools are adequate for evaluating the effect of global change on olive production. Models must be suitable for this task if they are to be applied to address questions related to climate change adaptation and/or mitigation. We will deal only with dynamic crop models which should include at least the simulation of phenology, growth and yield. Published models targeting the simulation of specific processes are also considered when they are mechanistic and present the potential to be used as components of full olive orchard models. This implies that empirical models are excluded from the review, even if they have been more common (e.g. Moriondo et al., 2015). We have structured the review in two main parts, the first related to the processes taken into account for the simulation of olive performance, i.e. the submodels, and the second to complete crop models of olive orchards. Both topics are preceded by a short introductory section on the most relevant uses of crop simulation models.

## 2 Utility of crop models for olive growing

### 2.1 Yield forecast

Forecasts of crop production provide independent and timely information to support policy making regarding agricultural markets. Crop models have frequently been the central elements of yield forecasting systems (e.g. Van der Velde et al., 2019). Indeed, the development of crop models started for meeting the demand for yield forecasts for the US as a strategic tool (Jones et al., 2017). Although crop models have been refined and improved in robustness over time, their direct application in yield forecasting is still limited by the lack of data and limitations of models (Basso and Liu, 2019). This has led to the application of alternative, empirical models, with wide adoption of deep learning techniques (Van Klompenburg et al., 2020), which are outside the scope of this review and are probably not efficient for the task (Morales and Villalobos, 2023).

### 2.2 Plant breeding programs

Crop models have been a great asset for understanding genotype-environment interactions and for designing ideotypes *in silico* for annual species. For instance, Agüera et al. (1997) used the *OilcropSun* model (Villalobos et al., 1996) to show the gains in sunflower yield associated with high early vigor genotypes. This use of crop models should be more powerful for perennials as breeding programs are slower and experiments more costly. In olives, the juvenile period, i.e. the time from germination to first flower, is typically 3 years or more (e.g. Hammami et al., 2021) which slows down the selection process. On the other hand, the productivity of new olive genotypes should be evaluated at least until maximum productivity is achieved, which takes 6-7 years in modern superintensive orchards and much longer in other orchard types (León et al., 2007). Therefore, experimental evaluation of new cultivars is time consuming and requires large dedicated plots to ensure homogeneous conditions. Crop models may incorporate parameters that can be measured rapidly in single plants, which makes the whole process much faster and cheaper than a purely experimental approach.

### 2.3 Hydrology

As olive orchards are important components of agroecosystems around the Mediterranean they have attracted attention in terms of hydrology and management to control soil erosion (Vanwalleghem et al., 2011). These problems occur at rather long-time scales so we need to resort to models. Furthermore, models are required also for calculating irrigation requirements in olive orchards (Testi et al., 2006). Water balance models of olive orchards have been proposed with different approaches, from very simple to quite complex. For instance, Abazi et al. (2013) used crop coefficients to calculate olive transpiration, while Garcia-Tejera et al. (2017) developed a full-fledged model based on the Soil-Plant-Atmosphere-Continuum (SPAC). The limitations of the crop coefficient approach have been shown for fruit trees in general by Villalobos et al. (2013), including its expected change as atmospheric CO_2_ concentrations increase.

### 2.4 Carbon balance and mitigation capacity of olive orchards

Society is encouraging farmers to reduce CO_2_ emissions associated with crop production. For instance, the green objectives in the new Common Agricultural Policy of the European Union will drive important changes in olive growing, like the adoption of protective cover crops during winter. On the other hand, carbon credit markets are emerging as a new opportunity for income for farmers in exchange for capturing C (Northrup et al., 2021). If we consider the soil, C capture is a slow process so soil organic matter changes may take many years before they can be detected (Fowler et al., 2023). Because of the possibility of performing simulations of decades or centuries, some models represent an invaluable asset to evaluate long-temporal trends in soil C stocks for specific environmental conditions and cropping systems (Mairech et al., 2020). Accumulation of C in standing trees may be quite variable as it depends among other factors on water and nutrient availability (Nardino et al., 2013). Therefore, carbon capture cannot be quantified solely by measurements but requires models that integrate all aspects of the carbon balance (tree photosynthesis and respiration, heterotrophic respiration, understory (if any) photosynthesis and respiration) in response to environmental (radiation, temperature, water availability) and management (pruning, tillage) factors (López-Bernal et al., 2023).

### 2.5 Impacts of global change

Fruit tree plantations are long-term projects designed to last more than 15-20 years. Therefore, expected environmental changes will have an effect on the overall performance of the plantation. The analysis of the expected effects of global change on crop production has been mostly devoted to annuals (White et al., 2011). For fruit trees and vines, the analyses of productivity under climate change have been mostly qualitative (e.g. Keller, 2010). Most attention has been directed at phenological development as warming may threaten the accumulation of enough chilling during winter (De Melo-Abreu et al., 2004), while only a few studies have quantified changes in olive productivity (Mairech et al., 2021; López-Bernal et al., 2023). In addition, physiologically based demographic modelling (PBDM) approaches have been used for evaluating how yield losses due to the olive fruit fly pest may change under climate change scenarios for different regions (e.g. Gutierrez et al., 2009; Ponti et al., 2014).

## 3 Olive models and submodels

### 3.1 Phenological development

Predicting phase development of olive trees has emerged in the last 20 years as an important topic for researchers due to its potential to address many practical questions related to the thermal adaptation of this species under new environments or future climate scenarios. So far, most studies on olive phenology have focused on the timing of flowering while other stages of the reproductive and vegetative cycle have barely received attention.

Olive blossoms in late spring in Mediterranean regions. Inflorescence formation requires low temperatures, their initiation taking place at the end of winter on 1-year axillary buds upon exposure to chilling for a long enough period (Haberman et al., 2017). Incomplete chilling delays the release of floral bud dormancy and reduces inflorescence and fruit production (Engelen et al., 2023), and it can result in asynchronous bud break and flowering (Medina-Alonso et al., 2020) or even suppress the production of reproductive structures under very warm environments (Hartmann, 1953). After dormancy release, warm temperatures contribute to accelerating development (Ramos et al., 2018; Di Paola et al., 2021).

The mathematical description of flowering time in olive has often capitalized on existing models of spring phenology developed for temperate trees. Purely *thermal time* models are based on heat accumulation after a fixed date, and they provide the simplest alternative for predicting flowering time. Model accuracy can be quite high for local conditions if thermal time requirements are properly calibrated (e.g. Model 3 in De Melo-Abreu et al., 2004). On the contrary, in fixing the date at which thermal time summation starts (e.g. February 1^st^ in the Northern hemisphere) some empiricism is introduced, as it is implicitly independent of the time at which dormancy is actually released. This issue may not be a big deal in regions with cold winters, where average temperatures are close to or lower than the base temperature, so heat accumulation is negligible until late winter. Nevertheless, it makes this type of model inappropriate for warm locations and climate change studies.

*Sequential models* offer a more comprehensive simulation of the processes leading to flowering, which are divided into two stages. The first accounts for dormancy release as a function of chilling accumulation. After enough chilling has been accumulated (i.e. ‘chilling requirement’), the forcing phase starts, which simulates the developmental rate as a function of heat accumulation. A number of sequential models have been developed and/or calibrated for olive orchards in the last decades (**Table 1**), each one characterized by a particular combination of approaches for computing chill and heat accumulation. Inspired by the Utah model used in other temperate species (Richardson et al., 1974), piecewise approximations are typically applied for estimating the increment of chilling as a function of hourly or daily temperature (e.g. De Melo-Abreu et al., 2004). Reversal of chilling due to high temperatures may be considered as well. On the other hand, heat accumulation may be simulated from the difference between mean daily temperature and base temperature or use more complex, sometimes non-linear, temperature response functions at the hourly scale (e.g. Marra et al., 2018).

**Table 1:** Types of spring phenology models specifically developed or calibrated for predicting full flowering in olive trees.

Model	Reference	Type of model	Chill accumulation	Forcing phase
DMA-1	De Melo-Abreu et al. (2004)	Sequential	Piecewise	Linear GDD
DMA-2	De Melo-Abreu et al. (2004)	Sequential	Hours below 7°C	Linear GDD
DMA-3	De Melo-Abreu et al. (2004)	Thermal time		Linear GDD
GL-4	Gabaldón-Leal et al. (2017)	Thermal time^a^		Linear GDD
Utah+GDH	Marra et al. (2018)	Sequential	Piecewise	Non-linear GDH
UniChill	Moriondo et al. (2019)	Sequential	Sigmoid	Non-linear GDH
CAC+GDD	Didevarasl et al. (2023)	Sequential	Piecewise	Linear GDD
Phenoflex	Didevarasl et al. (2023)	Flexible	Dynamic model	Piecewise GDH

The first column shows the name of the model. When not available, the models have been named after the initial of the first author or indicating the acronyms of the chilling and heat accumulation submodels. Studies dealing with several models have as many entries as models. The last two columns provide an indication on the approach followed for estimating chill and heat accumulation, respectively. CAC, Chill Anti-Chill days model; GDD, growing degree days; GDH, growing degree hours. ^a^The model by Gabaldón-Leal et al. (2017) estimates whether chilling requirements are met, but heat accumulation is independently computed from a fixed date, so it works as a thermal time model to all effects.

The main problem with sequential models is related to the lack of empirical records delineating chilling requirements for olive trees. Indeed, values for different cultivars have been estimated by fitting model parameters against flowering date records (De Melo-Abreu et al., 2004). To some extent, this is undesirable because chilling and heat requirements are calibrated together, which challenges their accurate determination. Besides, the flowering response to chilling is not a Boolean function, so flowering may proceed following a rather warm winter even if chilling accumulation is not enough to meet the estimated requirements. This seems to be the case in warm olive growing areas (e.g. Medina-Alonso et al., 2020). This phenomenon also casts doubt on how well existing chilling sub-models mimic tree responses to temperature during dormancy.

*Parallel* and *alternating* models are more complex process-based models accounting for possible compensation between chill and heat accumulation, which implies that some chill beyond the minimum requirement reduces the amount of heat necessary for flowering, as observed for some temperate trees (Pope et al., 2014). Both modelling frameworks present a large number of parameters so extensive calibration datasets are required to avoid overfitting. To the best of our knowledge, neither parallel nor alternating models have been tested for olive so far, but a recent study (Didevarasl et al., 2023) has used a modelling framework with a flexible overlap of chill and heat accumulation. The so call *Phenoflex* model (Luedeling et al., 2021) couples the dynamic model for chill accumulation (Fishman et al., 1987a; Fishman et al., 1987b) with a sigmoidal growing degree hour sub-model (Anderson et al., 1986) and it includes 12 parameters. However, the higher complexity of *Phenoflex* does not necessarily translate into a substantial improvement in the prediction of flowering time with respect to sequential models (Didevarasl et al., 2023).

The simulation of other events of the reproductive cycle has received less attention. Sequential models are appropriate for predicting bud break dates (Cesaraccio et al., 2004), although *Phenoflex* has also been used (Didevarasl et al., 2023). Fruit development seems to be affected by exogenous (environmental conditions) and endogenous (crop load) factors (Beltrán et al., 2017). Thermal time approaches have led to satisfactory results in some cases (Trentacoste et al., 2012; Didevarasl et al., 2023), but they are often unable to match the length of fruit developmental phases (Di Paola et al., 2021).

With regard to vegetative development, trees stop growing in late fall and growth is resumed whenever favorable temperature conditions return in early spring. As this winter rest stage seems to be controlled by temperature, López-Bernal et al. (2020a) proposed two simple chilling accumulation models for estimating the date of the onset of vegetative dormancy. Vegetative bud break has been simulated by simple empirical rules so far. For instance, *OliveCan*, a full model of the development, growth and yield of olive orchards (López-Bernal et al., 2018), counts the days with a mean temperature above a threshold after the winter solstice and sets the end of the rest period after a certain number have been reached. Likewise, Moriondo et al. (2019) used a thermal time approach for the task, with heat accumulation starting on a fixed date (January 1^st^).

### 3.2 Water balance

The different components of the water balance of olive orchards have drawn the attention of numerous researchers, who have developed specific submodels. Evaporation from the soil surface below the trees may be evaluated using the equation of Bonachela et al. (1999), who also proposed a method for including evaporation from wet bulbs under drip irrigation (Bonachela et al., 2001). Models of olive transpiration have followed different approaches, from the simple use of a transpiration coefficient (Orgaz et al., 2006) to the coupling of conductance, photosynthesis and water potential (Garcia-Tejera et al., 2017; see 3.3.4). Villalobos et al. (2013) proposed a simple mechanistic-based equation for the calculation of transpiration of fruit trees and calibrated it for olive and other fruit species.

Rainfall interception in olive canopies was modeled by Gómez et al. (2001). Additional studies of infiltration in olive orchards led to a method for determining the curve number of olive orchards (Romero et al., 2007) which is used for calculating surface runoff with the Soil Conservation Service method (Boughton, 1989).

Direct evaporation from wet olive trees may be calculated using the Penman-Monteith equation for zero canopy resistance until all intercepted rainfall is evaporated (López-Bernal et al., 2018). The required aerodynamic resistance is deduced from the model proposed by Raupach (1994). The transpiration of the cover crop below olive orchards may be calculated as a function of intercepted radiation (López-Bernal et al., 2023) following the general procedure of *Ceres* models (Garrison et al., 1999).

### 3.3 Biomass accumulation and partitioning

#### 3.3.1 Simple models

Crop growth may be simulated using a photosynthesis model (see 3.3.2) or following a simple model based on resource use. Monteith (1977) showed that the biomass accumulation (ΔB) of crops is linearly related to intercepted radiation. Therefore, for a period of duration t:


(1) $$\Delta B=\text{RUE}\;\sum_{1}^{t}f_{\text{i}}\;R_{\text{spi}}\;$$

Where RUE is the Radiation-Use Efficiency (g dry matter (MJ PAR)^-1^), *R*_spi_ is incoming Photosynthetically-Active Radiation (PAR, MJ m^-2^) and *f*_i_ is the fraction of PAR intercepted on day ‘i’. Mariscal et al. (2000a) were the first to measure the RUE of olive trees and Mariscal et al. (2000b) proposed a model of radiation interception for olive canopies which is currently used in *OliveCan*.

Alternatively, some crop models like *Cropsyst* (Stöckle et al., 2003) have adopted water use as the basis for estimating biomass accumulation. The idea was originally developed by Tanner and Sinclair (1983), who showed that the Water-Use Efficiency (WUE), the amount of dry matter produced per unit of water transpired, is inversely proportional to Vapor Pressure Deficit (VPD). This idea has been applied to quantifying tree photosynthesis in olive orchards (López-Bernal et al., 2015a) as a function of transpiration. However, because of the response of photosynthesis to internal CO_2_ (see 3.3.3), WUE will increase in proportion to stomatal resistance (Brodribb, 1996). This problem is common to both WUE and RUE simple approaches.

#### 3.3.2 Photosynthesis and respiration

Early models of crop photosynthesis used empirical relationships between leaf CO_2_ uptake and irradiance which could be integrated for the whole canopy (De Wit, 1965). This approach was incorporated into the olive model of Abdel-Razik (1989). Mechanistic models of photosynthesis arrived much later with the work of Farquhar et al. (1980), who provided equations for predicting the effects of temperature, radiation and internal CO_2_ concentration on leaf photosynthesis. The parameters of this model were measured for olive by Diaz-Espejo et al. (2006) and later included in *OliveCan* and the model of Morales et al. (2016).

Scaling up of leaf to tree photosynthesis requires a distribution of radiation among leaves in the canopy. Morales et al. (2016) used the approach of *Maespa*, which calculates radiation at a set of points within the crown. Calculation of photosynthesis is then performed at each point and the values are integrated. On the other hand, in *OliveCan* we followed the method of Mariscal et al. (2000a) that performs the calculation only at the soil level, then deduces intercepted radiation and sunlit leaf area and calculates photosynthesis for the two classes (sunlit and shaded leaves) following De Pury and Farquhar (1997).

The model of Farquhar assumes steady-state conditions so it ignores rapid fluctuations in light and temperature that occur within the canopy. To address that situation, a dynamic photosynthesis model is required (e.g. Morales et al., 2018) although it has not been tested in fruit trees so far.

Respiration models still follow the division into maintenance (proportional to biomass) and growth (proportional to photosynthesis) components proposed by McCree (1970). That idea was later completed with the works of Penning de Vries (De Vries et al., 1974; De Vries, 1975), which provided a mechanistic biochemical basis for the simulation of the two components. Pérez-Priego et al. (2014) calibrated the maintenance respiration parameters of the different organs of olives (stems, leaves, fruits) and measured their response to temperature. Mariscal et al. (2000a) determined the coefficients of growth respiration for olives. McCree and De Vries approaches have been incorporated into the olive models of Morales et al. (2016) and *OliveCan* (López-Bernal et al., 2018).

The response of photosynthesis to increased CO_2_ concentration seems to be limited by the inability of plants to use the excess C (downregulation of photosynthesis by sink limitation) (Ainsworth et al., 2004). However, the existing evidence in olive trees (Tognetti et al., 2001) shows almost no downregulation, although some differences may exist among cultivars. A lack of downregulation has been observed in many other tree species (Herrick and Thomas, 2001; Davey et al., 2006). Besides, evidences of downregulation in some forest trees under enriched CO_2_ seems to be the result of reductions in leaf N concentration (Medlyn et al., 1999). Therefore, N availability may limit the response of olive trees to higher CO_2_ and deserves specific research.

#### 3.3.3 Conductance and transpiration

The model of photosynthesis of Farquhar sets a biochemical limit for CO_2_ uptake by the leaf (demand) but does not yield directly the actual photosynthesis. The latter is the result of an equilibrium between the calculated CO_2_ demand and the supply, which is the flux of CO_2_ entering via stomata, i.e. the product of stomatal conductance and difference in CO_2_ concentration between the atmosphere and the leaf mesophyll (Leuning, 1995). Stomatal conductance is proportional to CO_2_ concentration and air humidity. The first to propose such a model were Ball et al. (1987), who used CO_2_ concentration at the leaf surface and relative humidity. Later, Leuning (1995) modified it to consider internal CO_2_ concentration and VPD. Moriana et al. (2002) calibrated the model of Leuning for olives and showed its advantage over previous empirical models like that of Stewart (1988), which had been used by Villalobos et al. (2000) for that species. The model of Leuning has been used in *Maespa* (Duursma and Medlyn, 2012) which serves for different tree species and was adapted for olives by Morales et al. (2016). Both, Leuning (1995) and Ball et al. (1987) used net photosynthesis as a driver for stomatal conductance.

Dewar (2002) opted for gross (instead of net) photosynthesis as the driving variable of conductance, so minimum (nighttime) conductance occurs when gross photosynthesis is zero. Tuzet et al. (2003) incorporated the role of leaf water potential in stomatal closure, which superseded the role of air humidity. However, in Tuzet’s approach, the calculation of conductance requires the simultaneous determination of leaf water potential (see 3.3.4). Fortunately, the high atmospheric coupling of olive trees (Villalobos et al., 2000) simplifies the solution, as boundary layer resistance does not need to be considered. This approach was adopted by López-Bernal et al. (2018) for the calculation of photosynthesis in OliveCan.

#### 3.3.4 Water uptake

Two distinct approaches have been employed to model water uptake: macroscopic and microscopic. The macroscopic approach describes root system water uptake using empirical functions that respond to water potential (Feddes and Raats, 2004). Such models have been extensively employed by soil hydrologists to calculate the sink term in Richard’s equation (e.g. *Hydrus* model, Šimunek et al., 2012). Conversely, the microscopic approach involves upscaling water uptake from a single root to the entire root system. Most models used for calculating water uptake by individual roots employ the analytical solution proposed by Gardner (1960), which calculates the water uptake of a single root by considering the water potential difference between the midpoint of two consecutive roots and the root surface, divided by the soil hydraulic resistance.

Since Gardner’s groundbreaking solution, research on soil-root interactions has incorporated additional factors that affect water flow paths. Herkelrath et al. (1977) included the effect of root contact, while Bristow et al. (1984) explored the interaction between soil texture and rhizosphere resistance. Later on, other researchers kept exploring drought effects on roots. North and Nobel (1997) and Stirzaker and Passioura (1996) delved into the impact of dryness on the contact between roots and soil and proposed conceptual models. North and Nobel (1992) investigated the effects of dry conditions on root morphology, such as suberification and collapse of the root cortex. They demonstrated the decay and recovery of root radial hydraulic resistance during dry and wetting cycles. Finally, Steudle and Peterson (1998) provided a more detailed description of water flow paths through the root, introducing the concept of ‘composite transport’. The composite transport model distinguishes three water paths in roots: the symplastic path (across cell walls), the apoplastic path (around cells), and the transcellular path (through cells). This model acknowledges the roles of hydraulic and osmotic forces in water uptake.

Recent advances in root water uptake modelling are linked to the explicit representation of the root hydraulic architecture (RHA), (like *R-SWMS*) (Lobet et al., 2014). These models represent a significant step towards a more comprehensive understanding of root water dynamics. The RHA models combine a three-dimensional (3D) representation of the root system with transport equations for individual roots (Doussan et al., 1998). This approach allows for capturing the uneven distribution of roots in the soil and the variation in water uptake rates. This becomes particularly important in scenarios involving drip or micro sprinkler irrigation, where contrasting soil moisture conditions and root densities are generated (Fernández et al., 1991; Fernandes et al., 2021). While most of the developments in RHA models have focused on annual crops (primarily maize), efforts have also been made in the context of trees. For instance, Vercambre et al. (2003) developed a 3D representation of plum root architecture to investigate water and nutrient uptake. In the case of olive trees, Sorgonà et al. (2018) described coarse root architecture on a high-density orchard.

**Figure 1:**
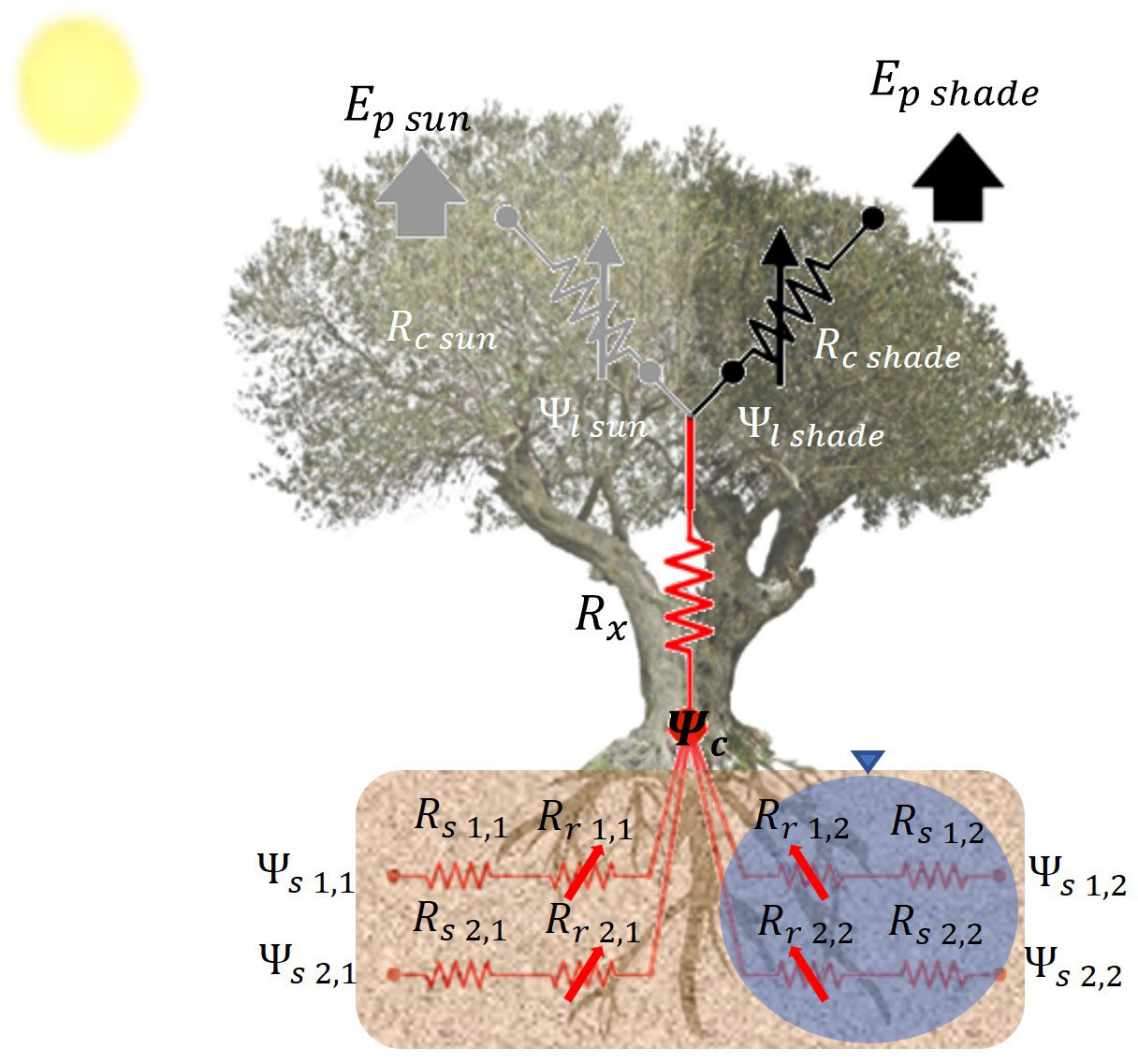
Schematic representation of the SPAC model with a soil multicompartment solution. The diagram represents the transport for water from the two soil compartments (irrigated and non-irrigated soil fractions) to the atmosphere. Symbols in the diagram are the soil (*R_s_*), root (*R_r_*) and xylem (*R_x_*) resistances, the soil (*Ψ_s_*), collar (*Ψ_c_*) and leaf (*Ψ_l_*) water potentials, and the stomatal resistance (*R_c_*) and transpiration (*E_p_*) for sunlit and shaded canopy fractions. Numbers indicate the soil layer and the soil compartment. Arrows indicate that the resistance is variable.

The modeling of water uptake in olive trees is still in its early stages. Garcia-Tejera et al. (2016) and López-Bernal et al. (2020b) have conducted studies demonstrating that olive tree root radial hydraulic resistance varies with temperature, which is the primary cause of low water potentials during winter in Mediterranean climates (López-Bernal et al., 2015b). Garcia-Tejera et al. (2016) proposed an empirical model to account for such variation. To capture the effects of root system distribution on olive tree behavior under deficit conditions, Garcia-Tejera et al. (2017) developed a SPAC model, which incorporates a soil multicompartment solution (see **Figure 1**). This model lies between simple 1D models and a more detailed 3D representation of the root system. It divides the soil into two horizontal compartments and several vertical layers, allowing for the accommodation of differential soil wetted fractions induced by drip irrigation systems. The SPAC model has been integrated into *OliveCan* to simulate the effects of water stress under localized irrigation.

### 3.4 Yield

#### 3.4.1 Harvest index

Yield may be calculated as the product of biomass and Harvest Index (HI) (Donald, 1962). The value of HI may be taken as constant or alternatively assuming a linear increase with time since flowering (Bindi et al., 1999). The former approach was included in the olive model proposed by Moriondo et al. (2019), while the last one has not been tried in trees. A more elaborate approach is using partitioning coefficients, so biomass of the harvestable organ receives a fraction of available carbohydrates during a given period as in the model of Morales et al. (2016).

An even simpler approach for calculating olive yield is based on the concept of RUE for oil production (Villalobos et al., 2006), i.e. the amount of oil produced per unit intercepted PAR, which lies between 0.17 g oil (MJ PAR)^-1^ in intensive orchards (Iniesta et al., 2009) and 0.12 g oil (MJ PAR)^-1^ in hedgerow olive groves (Connor et al., 2016).

#### 3.4.2 Number of fruits

Olive and many other tree species for which there is no direct manipulation of fruit load (i.e. fruit thinning) exhibit alternate bearing i.e. years with high yields are followed by years with low yields, which is associated with changes in the number of flowers (Monselise and Goldschmidt, 1982). Olive fruits appear on inflorescences originating on shoots grown in the previous season. The experiment of Stutte and Martin (1986) showed that manipulation of the number of fruits at the start of the summer affects flowering in the next season. This led to the idea of a so-called “floral induction” in summer which has been challenged since the work of Dag et al. (2010). This is so because new lateral buds are being formed until mid-autumn and they can potentially bear inflorescences in the next spring. Nevertheless, all of the buds on well-lignified parts of the shoot can potentially differentiate to form inflorescences (Lavee, 2015).

As new inflorescences appear on previous years’ generated buds the availability of carbon for new shoot growth limits the potential number of inflorescences that can be formed for the next season. Heavy fruit load implies reduced shoot growth which will be ultimately the reason for alternate bearing (Connor and Fereres, 2005). The equilibrium between vegetative and reproductive growth of the tree may be broken by any factor leading to low fruit numbers such as extreme water deficit around flowering (Rapoport et al., 2012) or lack of winter chilling (Hartmann, 1953; De Melo-Abreu et al., 2004). Other possible causes are poor pollination and reduced fruit setting due to heat (Koubouris et al., 2015), or fruit drop in later stages e.g. by pest attacks (Perdikis et al., 2009). All in all, any imbalance between vegetative and reproductive growth will mark the start of oscillations in fruit number and thus, yield.

To analyze the interaction between tree growth and the number of fruits we can use a conceptual model as follows. Yield (*Y*, g m^-2^) is the product of mean fruit weight (*w*, g glucose/fruit) and the number of fruits (*N*_f_, m^-2^), which may be calculated as a function of the number of positions (leaf axils) (*N*_p_, m^-2^):


(2) $$Y=N_{\text{p}}\;\alpha \;\beta \;w\;$$

where α is the ratio number of inflorescences per position (dimensionless) and β is the number of fruits per inflorescence (dimensionless).

A fraction (*f*_s_) of tree photosynthesis (*P*, g glucose m^-2^) is directed to shoot (including fruit) growth. The production of each new leaf position requires an amount of glucose γ (g). Therefore, the number of positions generated in a given year may be calculated as a function of the number of positions in the previous season (*N*_p_’), the fraction (*f*_s_) of *P* directed to the shoot and γ:


(3) $$N_{\text{p}}=\frac{f_{\text{s}}\;P-N_{\text{p}}^{'}\;\alpha \;\beta \;w}{\gamma }\;\;$$

By combining equations 2 and 3, yield may be expressed as a function of the previous year’s yield (*Y’*):


(4) $$\;Y=\frac{\;\alpha \;\beta \;w}{\gamma }(f_{\text{s}}\;P-Y')$$

**Figure 2:**
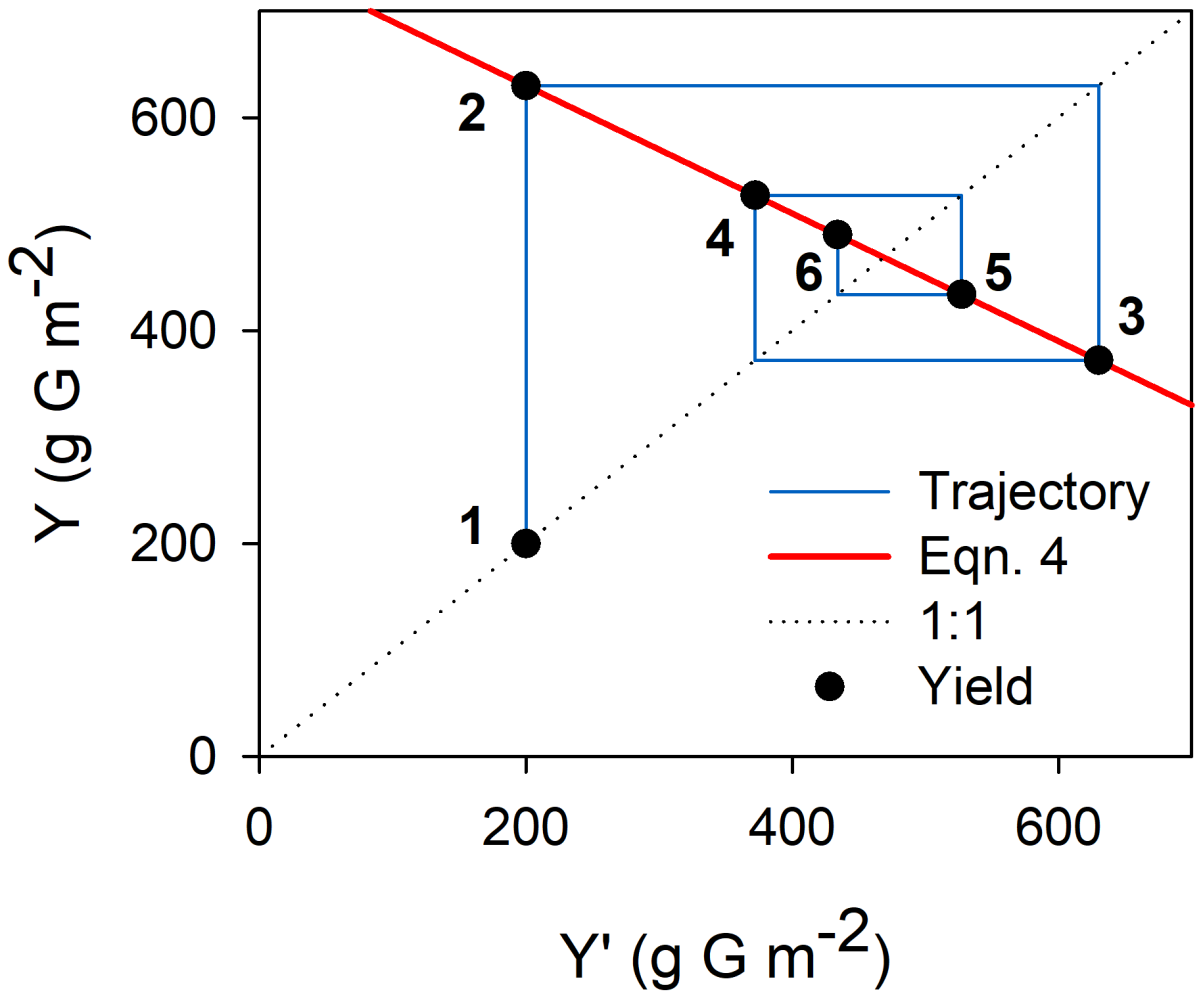
Graphical representation of the dampening of yield oscillations following a year of low yield due to an adverse exogenous event (e.g. severe water deficit during flowering leading to low fruit set and yield). Yield in successive years (solid circles numbered from 1-6) is calculated from Equation 4 (red line). ‘G’ in the axis titles stands for glucose. Parameter values for this example were *α*=0.5 inflorescences node^-1^, *β*=2.0 fruits inflorescence^-1^, *w*=1.5 g glucose fruit^-1^, *γ*=2.5 g glucose node^-1^, *f_s_* =0.5, P=2500 g glucose m^-2^. Initial yield was set as 200 g glucose m^-2^.


This recurrence equation provides a theoretical framework for understanding productivity in olive orchards. Mathematically, oscillations in yield dampen gradually if *α*·*β*·*w*/*γ* is lower than 1 and that must be the case in nature. Departures from equilibrium may occur due to exogenous events (e.g. lack of chilling, extreme weather events affecting fruit set), leading to oscillations that will tend to disappear over time until any further departure occurs (**Figure 2**)). The equilibrium yield (*Y*_eq_) is obtained when *Y* and *Y’* are equal so:

(5) $$Y_{\text{eq}}=\;\frac{f_{\text{s}}\;P}{1+\;\frac{\gamma }{\alpha \;\beta \;w}}$$

And equilibrium Harvest Index may be calculated as:


(6) $$HI_{\text{eq}}=\;\frac{1}{1+\;\frac{\gamma }{\alpha \;\beta \;w}}$$

Note that the definition of HI in trees has to be different from the classic one that we use in annual plants (ratio yield/shoot biomass) (Donald, 1962). For a perennial, HI may be defined as the ratio of yield and annual shoot growth.

Events reducing the number of fruits (e.g. heatwaves or drought at some stages) are likely to become more frequent in the future so departures from equilibrium yield and HI will be the rule. Specific submodels of fruit number will thus be required in olive crop models, while the use of HI or fixed partitioning coefficients to fruit (Morales et al., 2016) should be avoided unless the analysis goes beyond a single season (i.e. for the calculation of long-term average productivity).

The model encapsulated in eq. 4 shows that olive yield depends on tree photosynthesis (see section 3.3.2) and 5 parameters, namely:

- *f*_s_ (fraction of carbohydrates invested in the aerial part, i.e. not used in root growth): until now, the partitioning coefficients (PC) to coarse and fine roots have been taken as constants (Morales et al., 2016; López-Bernal et al., 2018). However, fine root turnover makes it difficult to accurately measure its PC (Soda et al., 2017). Furthermore, the root PC of trees increases in response to water stress (Ledo et al., 2018).
- α (number of inflorescences per position): its variation may be due to lack of chilling (Hackett and Hartmann, 1964; Malik and Bradford, 2005) or late harvest (Lavee, 2007) in “on” years. The chilling requirement for setting inflorescences is variable among cultivars (Hartmann and Porlingis, 1957).
- β (number of fruits per inflorescence): apart from clear genotypic differences (Lavee et al., 1996), the efficiency of flowering in olives may be reduced by poor pollination, fruit abortion or fruit drop. The first and second may be caused by water stress or heat stress during flowering (Koubouris et al., 2009; Fernandez, 2014). Pollination may be also prevented by a lack of compatible pollen (Sánchez-Estrada and Cuevas, 2018), which may occur in isolated monovarietal plantations or when rainy weather prevents airborne pollen transport (Rojo et al., 2015). After a few weeks from bloom, fruit drop will only occur under extreme water stress (Lavee et al., 1990).
- *w*: The average mass per fruit is, first of all, a cultivar characteristic that depends on cell number (Hammami et al., 2011). Fruit number will also affect final fruit size due to competition for available carbohydrates (Trentacoste et al., 2010). This effect would tend to reduce oscillations in yield. Nevertheless, it is clear that modelling olive yield in specific seasons requires both knowing fruit number and tree photosynthesis, i.e. we may be source or sink-limited.
- γ: the amount of glucose per leaf axil (position) is related to internode length, which may be variable among cultivars and depends on fruit load (Rosati et al., 2018), being higher in “off” years. This effect would also reduce oscillations in fruit numbers.

As a final remark, some authors have shown reductions in olive tree transpiration when fruit numbers are low (Bustan et al., 2016; Miserere et al., 2019), but the effect on tree photosynthesis is still unknown.

#### 3.4.3 Oil accumulation

Several studies have shown the typical patterns of oil accumulation for the main olive cultivars (Trentacoste et al., 2010; Navas-Lopez et al., 2019), finding a general negative effect of high temperature on oil concentration. The quality of olive oil is partly dependent on fatty acid composition which has been characterized by Rondanini et al. (2014) or Dag et al. (2011), who have shown a strong effect of harvest date and cultivars. However, despite the importance of oil concentration and quality on marketable price (Finotti et al., 2007), they have not been incorporated into existing olive crop models.

### 3.5 Soil carbon

Inputs of carbon to the soil include tree residues from senescence (leaves and roots) and pruning and the remains of cover crops (shoots and roots). In *OliveCan*, the heterotrophic respiration is calculated following Huang et al. (2009), including the effect of soil water content on decomposition (Verstraeten et al., 2006). Other soil carbon models are available but have not been incorporated to olive crop models. For instance, Nieto et al. (2019) applied *RothC* to evaluate the impact of changes in olive residue management.

### 3.6 Impacts of pests and diseases

Plant pathogens and crop-feeding arthropods are key components of agroecosystems. Simulating yield losses associated to these biotic factors requires a deep understanding of the complex interactions between the crop and its natural enemies, which are modulated by environmental conditions and management. As a result, the development of process-based models predicting the impacts of pests and diseases is still a major scientific challenge even for some of the most important crops (Donatelli et al., 2017). In olive trees, a PBDM approach has been proposed for simulating yield losses caused by the olive fruit fly (Gutierrez et al., 2009; Ponti et al., 2013; see below for more details).

### 3.7 Full models

A crop simulation model aiming to simulate the impacts of global changes should at least include submodels for phenological development, growth and yield. We will thus leave out the models proposed by Abdel-Razik (1989) and Viola et al. (2012) as well as *AdaptaOlive* (Lorite et al., 2018). The former two do not simulate phenology while the latter lacks a ‘growth’ model component.

Gutierrez et al. (2009) presented a PBDM approach for simulating the impacts of olive fruit fly on olive yield. In the model, fruit mortality due to fly attack is computed by considering fruit age and availability, so, although the emphasis is on the interactions between the olive trees and fruit fly populations, the model also includes components simulating flowering date and age-structured growth and yield of the trees. The effect of temperature on olive fly’s vital rates is also accounted for. This model has been subsequently refined by including a module of the water balance that accounts for the impacts of water deficit on photosynthesis rates (Ponti et al., 2013).

Morales et al. (2016) presented a model of olive orchards based on *Maespa* (Duursma and Medlyn, 2012), which in turn is an evolution of *Maestra* (Medlyn, 2004). It was designed for simulating potential growth and includes a joint photosynthesis-conductance model (FBBL type) and the phenology model of De Melo-Abreu et al. (2004). It uses fixed partitioning coefficients including one for fruits, that will determine yield unless flowering fails in case of insufficient chilling in winter.

López-Bernal et al. (2018) developed *OliveCan*, which includes the model of Mariscal et al. (2000b) for radiation interception and the model of De Melo-Abreu et al. (2004) for phenology. It combines an FBBL with the model of Tuzet et al. (2003) which is based on leaf water potential. The latter is simulated by solving the SPAC model proposed by Garcia-Tejera et al. (2017), driven by a full water balance submodel. It is thus the only mechanistic model of olive orchards in terms of response to water deficits. Furthermore, the model incorporates a submodel of the carbon and water balance of cover crops (López-Bernal et al., 2023).

Finally, Moriondo et al. (2019) introduced a much simpler olive model than the aforementioned. It uses the UniChill model (Chuine, 2000) for phenology, while biomass accumulation is simulated through a simple radiation-use efficiency approach. Yield is deduced by applying a HI value that can be reduced by various abiotic stresses.

## 4 Discussion

Crop models designed to cope with global change effects require a mechanistic and accurate description of tree responses to the increases in temperature and CO_2_ concentration. Furthermore, projections show also reduced rainfall around the Mediterranean (Longobardi and Villani, 2010), where most olive growing occurs, and an increase in climatic extremes (Diffenbaugh et al., 2007). However, the reported increase in extreme heat events seems to be the result of a shift in the mean value, and not in the variance (Simolo et al., 2014).

Many important processes are affected by temperature, including phenological development, respiration, photosynthesis, growth and senescence. The most dramatic effects of gradual temperature changes will be the change in flowering date and the possible lack of winter chilling. Episodic heat events may reduce pollination and fruit set. In any case, the need for accurate submodels of phenology and fruit number cannot be underrated as opposed to using HI, which can only be applied to gross long-term evaluations of productivity.

On the other hand, CO_2_ concentration affects photosynthesis and stomatal conductance. Higher CO_2_ improves potential photosynthesis but promotes some stomatal closure. The overall effect on tree carbon accumulation would depend also on the expected increase in respiration rate (due to higher temperature). Therefore, the efficiency parameters normally used to estimate biomass accumulation (RUE or WUE) will inevitably change in parallel to the atmospheric CO_2_ concentration, which forces the adoption of a photosynthesis/conductance model.

Available olive crop models have already incorporated satisfactory solutions for tree photosynthesis and conductance but there is still room for improvement. First, calibration of the parameters of the model of Farquhar et al. (1980) should be performed for the main olive cultivars and their dependence on leaf N concentration should be quantified. Second, inhibition of carboxylation by combined water and heat stress should also be considered. Third, the interactions of stomatal conductance, transpiration and water potential are still a work in progress. A more mechanistic approach to this problem requires a detailed model of the stomatal functioning in response to water potential in guard and accompanying cells (Buckley, 2017). Moreover, the inclusion of more mechanistic functions to simulate root water uptake under deficit conditions is a must if we aim to properly model olive behavior under the water stress conditions that the overwhelming majority of olive groves endure every summer. At moderate soil water deficits, stomatal closure is not driven by xylem cavitation but by the declines in soil water potential (Carminati and Javaux, 2020; Corso et al., 2020). Dietrich et al. (2018), demonstrated that cutting half of the tree sapwood had no effect on water potential nor in transpiration. In olive cultivar ‘Kalamata’, Rodriguez-Dominguez and Brodribb (2020) showed that 81% of the whole hydraulic plant resistance under non-stressed conditions belongs to root radial hydraulic resistance, and the value increases up to 95% under moderate water stress. Thus, efforts should concentrate on disentangling root water uptake response under water deficit. In maize, circadian oscillation in root radial hydraulic resistance has been observed, being proportional to previous water stress conditions (Caldeira et al, 2014). Does olive roots respond in the same way? The answer could reveal novel approaches to improve olive performance under water scarcity scenarios. For instance, simulations have shown that changes in root morphology could enhance water use efficiency in olives under deficit irrigation by developing rootstocks with more active root growth in the wet bulb (Garcia-Tejera et al., 2018). It is, therefore, crucial to advance in the development of more comprehensive models that link the water demand (canopy) and supply (roots) functions.

In terms of carbon allocation, available models incorporate partitioning coefficients that have only been measured in large trees (Villalobos et al., 2006) for the cultivar ‘Arbequina’. Apart from expanding the calibration to other cultivars with contrasting growth habits (e.g. ‘Frantoio’), we need to understand how water stress changes the partitioning of assimilates, with special emphasis on root/shoot C distribution, a delicate variable that may lead to significant errors over multiannual simulations. On the other hand, the modelling of reserve dynamics is still very simplistic in the main existing models, serving mainly as a pool for carbon not used in the growth of other organs. However, the work of Bustan et al. (2011) showed that reserves in olive trees act as a separate sink, i.e. some C is partitioned to reserves even when C supply is limited. More studies focused on the way olives distribute their assimilates in response to the environment are paramount to improve models, in particular their ability to simulate extreme conditions and multi-annual effects on growth.

Olive spring phenology models have been tested successfully for predicting flowering dates in traditional growing areas (De Melo-Abreu et al., 2004), where chilling requirements are always satisfied, but more complex approaches may be required in new environments of the Southern Hemisphere with warmer winters (Torres et al., 2017) and for the future projected climate around the Mediterranean. In any case, a better understanding of the requirements and responses to chilling is of the utmost importance to improve the robustness of existing and new models in the context of climate change studies.

The requirement of a submodel for fruit number in olive trees is amplified by the expected effects of warming, caused by the lack of winter chilling and/or by heat events during flowering. On the other hand, oil accumulation may be compromised by higher temperatures. The effects of C supply and temperature and their interactions on oil quantity and quality (fatty acid composition and organoleptic or stability compounds) should be incorporated into olive crop models if we want to evaluate the economic sustainability of orchards.

Finally, olive crop models will need to include an N balance submodel to improve the simulation of photosynthesis and the dynamics of organic dry matter, which determines the capacity for CO_2_ capture in the soil. Although Abdel-Razik (1989) introduced simple fertilization effects on growth, mechanistic models have overlooked olive N balance. A reason for this may be that N has rarely represented a concern in olive, due to the extensive management and the relatively low requirements of the traditional growing systems. Nevertheless, the new intensive and superintensive olive farming set a new scenario where N supply becomes quickly a relevant limitation to growth, and it is sometimes purposely part of the growth regulation techniques to extend the orchard lifespan. Assuming permanent N sufficiency in high demand systems may lead to significant mismatching in the calculation of growth and yield, both in long- and short-term simulations. The inclusion in the modern process-based models of a sound N uptake and distribution submodel -including N fate in soil organic matter- is paramount to correctly simulate olive growth and, especially, the increased photosynthesis rates expected under the future atmospheric CO_2_ levels.

## Author contributions

All authors listed have made a substantial, direct, and intellectual contribution to the work and approved it for publication.
